# Supplementary material for: Providing choice of feedback affects perceived choice but does not affect performance
Source: PeerJ. 2022 Jun 28;10:e13631. doi: 10.7717/peerj.13631 (PMC9248777; doi:10.7717/peerj.13631)
Supplement: Supplemental Information 2 [file peerj-10-13631-s002.docx]

Codes for categorical data in raw data file (also added to the raw data file in a second worksheet):

Group_original:

1 – offline/summary feedback

2 – online feedback

3 – autonomy group / choice of feedback

Choice:

1 – offline/summary feedback

2 – online feedback

Sex:

1 – male

2 – female

Group_yoked:

1 – autonomy

2 – yoked
